# Exosome-Based Cancer Therapy: Implication for Targeting Cancer Stem Cells

**DOI:** 10.3389/fphar.2016.00533

**Published:** 2017-01-12

**Authors:** Jinheng Wang, Yongjiang Zheng, Meng Zhao

**Affiliations:** ^1^Department of Hematology, The Third Affiliated Hospital, Zhongshan School of Medicine, Sun Yat-Sen UniversityGuangzhou, China; ^2^Key Laboratory for Stem Cells and Tissue Engineering, Ministry of Education, Sun Yat-Sen UniversityGuangzhou, China; ^3^Department of Pathophysiology, Zhongshan School of Medicine, Sun Yat-Sen UniversityGuangzhou, China

**Keywords:** exosomes, nanocarrier, cancer therapy, cancer stem cells, exosomal engineering

## Abstract

Drug resistance, difficulty in specific targeting and self-renewal properties of cancer stem cells (CSCs) all contribute to cancer treatment failure and relapse. CSCs have been suggested as both the seeds of the primary cancer, and the roots of chemo- and radio-therapy resistance. The ability to precisely deliver drugs to target CSCs is an urgent need for cancer therapy, with nanotechnology-based drug delivery system being one of the most promising tools to achieve this in the clinic. Exosomes are cell-derived natural nanometric vesicles that are widely distributed in body fluids and involved in multiple disease processes, including tumorigenesis. Exosome-based nanometric vehicles have a number of advantages: they are non-toxic, non-immunogenic, and can be engineered to have robust delivery capacity and targeting specificity. This enables exosomes as a powerful nanocarrier to deliver anti-cancer drugs and genes for CSC targeting therapy. Here, we will introduce the current explorations of exosome-based delivery system in cancer therapy, with particular focus on several exosomal engineering approaches that have improved their efficiency and specificity for CSC targeting.

## Introduction

Cancer remains one of the leading causes of death due to the late diagnosis, poor prognosis, and frequent occurrence of drug resistance and metastasis (Colak and Medema, 2014). Cancer stem cells (CSCs) are a small subpopulation of immortal cancer cells, capable of long-term self-renewal, and differentiation into heterogeneous cancer cell lineages (Bandhavkar, 2016). Although the existing of CSCs is still controversial, recently a number of studies have identified CSCs in several types of solid tumor, such as renal cancer, breast cancer, lung cancer, liver cancer, prostate cancer, melanoma, and in leukemia (Huang and Rofstad, 2016). CSCs are known as the source of primary and metastatic cancer, and the root of chemo- and radio-therapy resistance (Sales et al., 2007; Colak and Medema, 2014). Multiple mechanisms are involved in CSC drug resistance, including slow cell cycle progression, drug efflux, enhanced DNA repair efficiency, elevated anti-apoptotic capacity, and detoxification enzyme expression (Marhaba et al., 2008; Saha et al., 2012; Colak and Medema, 2014; Sotiropoulou et al., 2014; Lu et al., 2016). This combination of factors leads to the treatment failure and relapse frequently observed in cancer patients. Therefore, developing novel strategies to specifically target CSCs and overcome their drug resistance is pivotal for cancer treatment.

Nanotechnology-based drug delivery systems are a variety of synthetic nanoparticles and biological vesicles which have biological specificity for *in vivo* targeting therapies. In recent years, some synthetic nanoparticles have been employed as vehicles to deliver therapeutic drugs to the bulk of the tumor, and even directly target CSCs (Lu et al., 2016). Nanoparticles also have slow drug-releasing characteristics which induce a sustained high local drug concentration around the tumor and an enhanced anti-cancer efficiency (Ahmad et al., 2016; Piktel et al., 2016). As recently reviewed by Lu et al. several synthetic nanoparticles, such as liposomes, niosomes, micelles, polymeric, and gold nanoparticles are able to deliver anticancer drugs to target tumor cells; this precision is made possible by their ability to use CSC specific markers such as CD44, CD90, and CD133 to target a specific population. Furthermore, the specificity of such particles is enhanced by the use of different payloads which can inhibit specific signaling pathways including Notch, Hedgehog, and transforming growth factor-β (TGF-β) in CSCs (Lu et al., 2016).

Biological vesicles are naturally derived from bacteria, erythrocytes, or mammalian cells (Soltani et al., 2015). Bacterial ghosts are obtained from chemically inactivated Gram-negative bacterial cells after removal of their cytoplasmic contents. Bacterial ghosts can be used as a carrier for genes, drugs, and vaccines; however their lipopolysaccharide-caused immune responses have limited their use *in vivo* (Kudela et al., 2005, 2008, 2011; Mayr et al., 2005; Paukner et al., 2005). Erythrocyte ghosts are cytoplasmic-content free erythrocytes and have high biocompatibility and biodegradability. They are non-toxic and non-immunogenic with a long life span in circulation. Unfortunately their capacity for drug loading is limited, and deformations during transportation frequently cause unstable encapsulation and drug leaking, limiting their clinical use (Magnani et al., 2002; Muzykantov, 2010; Biagiotti et al., 2011; Yousefpour and Chilkoti, 2014). Exosomes, secreted from living cells, have been used as nanometric vehicles for therapeutic drug and gene delivery. They are biocompatible, non-cytotoxic, low immunogenic, simple to produce, easy to store, have a long life span, and high cargo loading capacity (Munagala et al., 2016; Srivastava et al., 2016; Wang et al., 2016b). These characteristics make exosomes a promising drug carrier for cancer treatment (Tian et al., 2013; Tang et al., 2015; Pitt et al., 2016). In this review, we provide an overview for exosome studies with a particular emphasis on current advances of exosome-mediated cancer targeting therapy.

### Characteristics of exosomes

Besides engaging in cell-cell contact and directly releasing soluble molecules through those interactions, extracellular vesicles (EVs) derived from cells also mediate the short-range and distant communications between cells (Hwang, 2013; Wang et al., 2014). EVs directly shed from the plasma membrane are heterogeneous particles with the size range of 100–1000 nm in diameter (van der Meel et al., 2014; Vader et al., 2016). Exosomes are derived from intracellular late endosomes but with a smaller size of 40–100 nm. Exosome formation is initiated by early endosomes, followed by the formation of intraluminal vesicles (ILVs) inside the endosomes. These endosomes enclosed within mature ILVs are called multivesicular bodies (MVBs), which can either fuse with lysosomes for degradation and recycling, or release ILVs as exosomes into the extracellular matrix through fusing with plasma membrane (Théry et al., 2002; Kharaziha et al., 2012; Klumperman and Raposo, 2014).

Exosomes contain receptors on their lipid bilayer membrane and carry proteins, lipids, mRNAs, miRNAs, and small DNA fragments inside to protect them from degradation (Raimondo et al., 2011; Hwang, 2013; De Veirman et al., 2016; Wang et al., 2016a). Exosomes can be distinguished by size and specific surface markers including TSG101, Alix, Flotillin-1 CD63, CD9, among other EVs (Schorey and Bhatnagar, 2008; Soltani et al., 2015; Tang and Wong, 2015; Yu et al., 2015). Exosomes are present widely in various cell culture-conditioned media and body fluids including synovial fluid, saliva, urine, breast milk, semen, and blood. Thus, several methods have been developed to isolate exosomes from body fluids or conditioned supernatant, including differential ultracentrifugation, density gradient centrifugation, size exclusion chromatography, immunoaffinity capture, and polyethylene glycol-mediated precipitation (Tauro et al., 2012).

### Function of exosomes

As a communicator, exosomes can directly stimulate multiple types of target cells with their membrane molecules or deliver their contents into cells for direct influence (Camussi et al., 2011; Raposo and Stoorvogel, 2013). Exosomes are transferred from original cells to destination mainly through the circulating flow and thereafter localized in target area through binding their membrane molecules to target cell surface receptors for long range communication. Cancer mouse models and cancer patients have higher levels of EVs and exosomes in body fluids (Taylor and Gercel-Taylor, 2008; Ghosh et al., 2010; Benameur et al., 2013), implying the involvement of these particles in cancer progression. Indeed, accumulating evidence revealed that exosomes play a critical role in tumorigenesis. For example, exosomes derived from mesenchymal stromal cells (MSC) or fibroblasts directly facilitate cancer progression and induce drug resistance in multiple myeloma, colorectal cancer, and gastric cancer cells through delivering various miRNAs and soluble factors into tumor cells (Roccaro et al., 2013; Wang et al., 2014; Hu et al., 2015; Ji et al., 2015). Astrocyte-derived exosomes transfer the miR-17~92 cluster to suppress PTEN gene in brain tumors (Zhang et al., 2015). Malignant cells also secrete large amount of exosomes to promote endothelial cell proliferation and enhance angiogenesis, which facilities tumor progression (Umezu et al., 2014; Wang et al., 2016a). Cancer cell-derived exosomes can also induce immunosuppression in the tumor microenvironment (Chalmin et al., 2010; Wang et al., 2016a). This tumor exosome-educated microenvironment in turn facilitates tumor survival and growth. Cancer cell-derived exosomes can preferentially fuse with the cells at their predicted destination to form a pre-metastatic niche for tumor metastasis (Hoshino et al., 2015). Furthermore, these cancer cell-derived exosomes can even convert normal epithelial cells to form tumors in mice (Melo et al., 2014). Acute myeloid leukemia cell-derived exosomes can deliver miR-155 into normal hematopoietic stem and progenitor cells (HSPCs), and suppress c-Myb expression to impair normal hematopoiesis, which in turn facilities leukemic cell growth (Hornick et al., 2016). All of these results underline the importance of exosome as a messenger for cell communication during cancer progression. Taking advantage of their natural delivery capability, exosomes have been successfully used for drug and functional RNA delivery vehicles in cancer treatment. Furthermore, an increasing number of researchers have devoted their efforts to improve the capacity, specificity, and selectivity of exosome-mediated nanodelivery in recent years.

### Advantages of exosomes for cancer therapy

Unlike synthetic nanoparticles, exosomes are more biocompatible and biodegradable, and thus have low toxicity and immunogenicity (Ha et al., 2016). Although other cell-derived EVs are also biocompatible, they are bigger than exosomes and more heterogeneous which limited their application for drug loading and delivery. Exosomes can also be easily generated because most cell types can produce exosomes. Exosomes are stable in biological fluids and their small size enables exosomes to easily escape from lung clearance and pass through the blood-brain barrier (Kawikova and Askenase, 2015; Li et al., 2016). Adherence and internalization of exosomes within tumor cells is 10-times higher than liposomes of a similar size, indicating a higher specificity of exosomes for cancer targeting (Smyth et al., 2014). In addition, due to enhanced permeability and retention effect, nanometric exosomes tend to accumulate in tumor tissues containing abnormally formed blood vessels than they do in normal tissues, thus exosomes can easily reach the bulk of the solid tumors to increase their drug delivery efficiency. Moreover, exosomes can be engineered with tumor-targeting proteins, peptides, or antibodies for precise drug and therapeutic nucleic acid delivery. Taken together, these characteristics make exosomes one of the best candidates for cancer targeting therapy.

### Exosome modifications for specific targeting

Synthetic nanoparticle-mediated delivery has low specificity because a very limited number of selective molecules can be used for cell targeting. However, natural cell-produced exosomes can recognize specific cell types via their surface receptors. For example, exosomes with Tspan8 preferentially bind to CD11b and CD54-postive cells (Rana et al., 2012). In addition, researchers can engineer donor cells to obtain modified exosomes with particular receptors for better cell recognition. Most exosome modifications are adapted from surface display technology which presents candidate proteins or peptides on exosome membranes. To achieve this, researchers engineer donor cells to express candidate proteins or peptides fused with an exosomal membrane proteins such as lysosome-associated membrane glycoprotein 2b (Lamp2b) and tetraspanins CD63 and CD9 (Stickney et al., 2016), which will position the candidates on exosome's surface. For example, dendritic cells (DCs) were engineered to express αv integrin-specific iRGD peptide and Lamp2b fusion protein, allowing the engineered DCs to secret exosomes with the iRDG peptide on their surface. These engineered exosomes have dramatically increased drug delivery efficiency and anti-tumor effect on αv integrin-positive breast cancer cells in a mouse model (Tian et al., 2014). Exosomes with the neuron-specific rabies viral glycoprotein (RVG) peptide can specifically bind to the acetylcholine receptor on neuronal cells and selectively knockdown certain genes in neurons, microglia, and oligodendrocytes by specific delivery of small interfering RNA (siRNA) into those cells (Alvarez-Erviti et al., 2011). These results indicated that adapted exosomes can serve as a powerful tool for neural cancer treatment. Furthermore, glycosylated peptides on the exosome's surface are resistant to proteasome-mediated degradation in circulation, which enhances their stability and even efficiency of targeted delivery (Hung and Leonard, 2015).

A recent study has used a magnet-based method to further improve the tumor targeting specificity. Qi et al. engineered magnetic exosomes by linking superparamagnetic-conjugated transferrin to transferrin receptor-positive blood exosome surfaces, and an external magnet was put on the tumor site *in vivo*; this allowed the magnetic exosomes to be directed to the target tumors cells to efficiently suppress tumor growth (Qi et al., 2016). Although the magnetic exosomes cannot directly target CSCs, the vast enrichment of exosomes loaded with potent CSC targeting drugs around solid tumors can significantly improve therapeutic efficiency and limit their side-effects by restricting drugs on tumor site. The anti-tumor specificity can be further improved by presenting specific anti-tumor antibodies on exosome surface. In one study, an engineered anti-epidermal growth factor receptor (EGFR) nanobody and exosome anchor signal peptide glycosylphosphatidylinositol (GPI) fusion protein were transfected to donor cells to generate nanobody-presenting exosomes. These exosomes were capable of directly targeted EGFR-positive tumor cells (Kooijmans et al., 2016).

Protecting drug-loaded exosomes from liver clearance is critical for their cancer treatment applications. Researchers blocked scavenger receptor class A family (SR-A), a monocyte/macrophage uptake receptor for exosomes, which dramatically reduced exosome liver clearance and enhanced their accumulation in tumor (Watson et al., 2016). Another approach involving exosome-liposome hybridization was also used to increase their specificity and stability (Sato et al., 2016). Nakase and Futaki utilized cationic lipids as “glue” to display pH-sensitive fusogenic peptides on exosome surfaces which enhanced their cell membrane binding and cell uptake efficiency of exosomes. After endocytosis, these peptides facilitated exosome and endosome fusion, which increased the releasing of exosomal cargos in cytoplasm (Nakase and Futaki, 2015).

**Figure 1:**
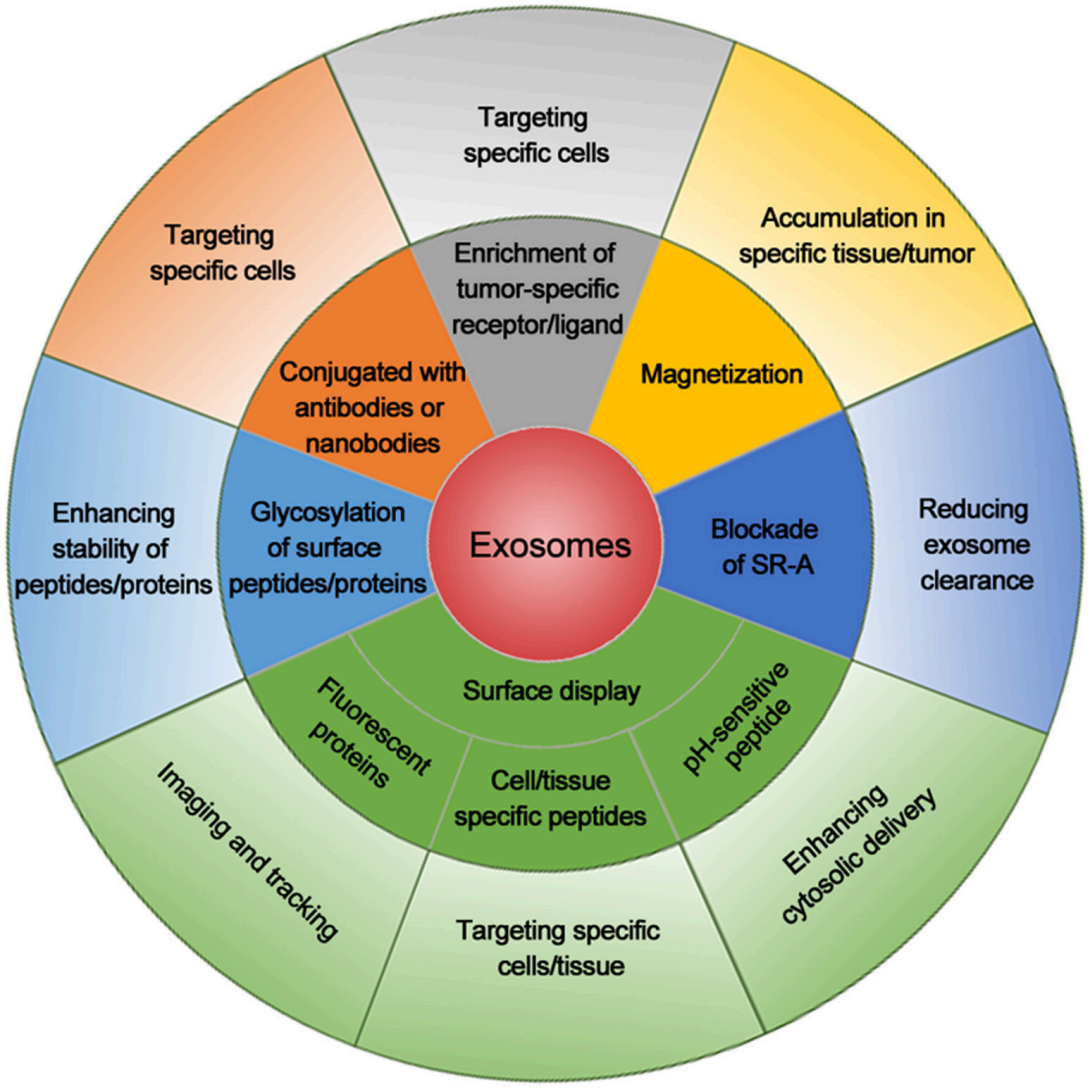
**Summary of exosomal modifications to enhance their cancer cell targeting efficiency**. Exosomes with cell/tissue-specific peptides, tumor-specific receptors/ligands, or antibodies/nanobodies for tumor markers increase their specificity for cancer cell targeting. Exosomes displaying fluorescent protein or chemical on the surface are used for imaging or tracking. Magnetization of exosomes elevates their accumulation around the tumor. Blockade of scavenger receptor class A family (SR-A) reduces the clearance of exosomes by liver and increases exosome concentration in circulation and tumor. Exosome linked with pH-sensitive peptide enhances the cytosolic delivery. Glycosylation of peptides/proteins on exosome surface increases the stability of exosomes and thus enhances their delivery efficiency. The combination of these methods will further enhance the delivery efficacy and specificity for cancer cell targeting.

Taken together, the success of these exosomal adapting methods, as summarized in Figure 1, provide an effective CSC treatment prospect and the combination of them will further improve the outcome of exosome-mediated CSC targeting.

### Exosome cargo loading for cancer therapy

As a delivery system, exosomes are widely used as vehicles for various tumor therapeutic cargos. The lipid bilayer membrane of exosomes forms a natural protective shelter and a sustained release capsule for various anti-cancer drugs. Various anti-cancer drugs and cancer gene suppressors, including functional RNAs, have been used for exosome based tumor treatment (Seow and Wood, 2009; Camussi and Quesenberry, 2013). To get the best tumor treatment effect, high efficacy exosome loading methods are critical. Small membrane-permeable agent loading can be achieved by incubating agents with exosomes (Hood, 2016). However, to load membrane-impermeable drugs, miRNAs, siRNAs, and small DNAs, electroporation is required to create pores on exosome lipid bilayer membrane to allow them to be encased within exosomes (Wahlgren et al., 2012; Hood et al., 2014; Momen-Heravi et al., 2014). Unfortunately, even though commercial membrane-permeable reagents such as liposomes have been used for assisting RNA and DNA fragment loading into exosomes, their efficiency did not satisfy most of the researchers (Wahlgren et al., 2012; Shtam et al., 2013). Pre-overexpression of candidate RNAs or proteins in donor cells is still considered as the best way to generate candidate protein- and RNA-loaded exosomes (Munoz et al., 2013).

### Anticancer agents

The best exosome cancer therapy is to have chemotherapeutic drug-loaded exosomes specifically targeting CSCs *in vivo*. Accumulating evidence has shown that exosome-mediated chemotherapeutic delivery has much improved anti-tumor effects when compared to free drugs in animal tumor models. For example, doxorubicin is a common chemotherapeutic drug to treat hematological malignancies and many types of solid tumors and sarcomas. Doxorubicin is fluorescent and easily tracked, therefore it has been well studied in exosome-mediated cancer therapy. In a colon adenocarcinoma mouse model, exosome-delivered doxorubicin shrank tumor size much more efficiently than did free or liposome-delivered doxorubicin (Jang et al., 2013). Furthermore, using αv integrin-specific iRGD peptide presenting exosomes to deliver doxorubicin dramatically enhanced the anti-tumor effect in αv integrin-positive breast cancer cells in animals compared to free drug administration (Tian et al., 2014). Notably, exosome-mediated doxorubicin delivery has dramatically reduced its cardiotoxicity, which is considered to be the major side effect of doxorubicin in clinical applications (Toffoli et al., 2015). This is due to the exosome-mediated restriction of doxorubicin from crossing through myocardial endothelial cells (Hadla et al., 2016). Due to the advantages of this delivery system, higher concentrations of doxorubicin can be used to treat breast and ovarian tumors while reducing off-target effects (Hadla et al., 2016). Overall, these studies suggest that exosome-mediated doxorubicin delivery has great prospects for clinical application.

Paclitaxel is another widely used antimitotic chemotherapeutic drug for various tumor therapy (Liu et al., 2015; Bakrania et al., 2016). Paclitaxel can be loaded into exosomes by sonication, and these loaded exosomes have 50 times more cytotoxicity than free paclitaxel for drug resistant cancer cells *in vitro*. They can also dramatically block murine Lewis lung carcinoma pulmonary metastases and reduce tumor size in the mouse model (Kim et al., 2016). This indicates that exosome-encapsulated paclitaxel can directly target drug resistant CSCs. Moreover, prostate cancer cell-derived exosomes loaded with paclitaxel also have enhanced cytotoxicity to autologous cancer cells (Saari et al., 2015). Interestingly, drug-pretreated donor cells can also produce drug-loaded exosomes. For example, exosomes derived from paclitaxel-treated MSCs exhibited a strong inhibitory effect on human pancreatic adenocarcinoma (Pascucci et al., 2014).

Withaferin A, a potent inhibitor of angiogenesis and cancer growth, has also been tested in exosome-mediated delivery therapy. Either intraperitoneal or oral administration of exosomes-loaded withaferin A showed a much stronger anti-tumor effect compared to free drugs in human lung cancer xenograft mouse model (Munagala et al., 2016). Exosomes loaded with celastrol, a triterpenoid derived from plants (Chang et al., 2003), also showed stronger anti-tumor effect compared to free celastrol in human lung cancer cell xenograft model (Aqil et al., 2016). Some hydrophobic anti-tumor drugs such as curcumin, can be easily incorporated into exosome surfaces through the lipid bilayer membrane binding (Sun et al., 2010; Hood, 2016), which qualifies them as potential candidates for exosome-mediated drug delivery.

### Peptides and proteins

Besides anti-cancer therapeutic drugs, exosomes can also deliver various tumor antigens (Cho et al., 2005), apoptosis-inducing proteins (Hall et al., 2016), nanobodies (Kooijmans et al., 2016), deficient or mutant anti-apoptosis proteins (Aspe et al., 2014), tumor and tissue-specific peptides (Hung and Leonard, 2015), proteasomes (Lai et al., 2012), transferrins, and lactoferrins (Malhotra et al., 2016) into cancer cells for targeting therapy.

DCs are widely used for T cell-mediated immunotherapy by presenting tumor antigens to naive T cells, however this strategy is limited by the short life span of DCs after activation (Hermans et al., 2000). Nevertheless, researchers found that exosomes derived from peptide-pulsed DCs, can present antigens to T cells to induce their immune response. These DC-derived exosomes contain MHC-peptide complexes and co-stimulatory molecules on their membrane, which enable them to prolong antigen presentation and boost immunization in mice compared to antigen-presenting DCs (Luketic et al., 2007). Furthermore, exosomes isolated from two MHC type-distinct mouse cell lines expressing tumor antigen human mucin 1 (hMUC1), induced an effective immune response and suppressed hMUC1-expressing tumor cell growth in mice (Cho et al., 2005). Exosomes obtained from malignant mesothelioma cells were found to contain tumor antigens; administration of these exosomes can improve the overall survival of mesothelioma-bearing mice by activating anti-tumor immune responses (Mahaweni et al., 2013).

Survivin, an anti-apoptotic protein, plays important roles in multiple cancer cells to suppress apoptosis activation. Inactive mutation survivin-T34A, impair its pro-survival activity and induce caspase activation and apoptosis in cancer cells (Aspe and Wall, 2010). Survivin-T34A-loaded exosomes can induce apoptosis in various pancreatic adenocarcinoma cell lines, and enhance their sensitivity to gemcitabine (Aspe et al., 2014). Natural cell-derived exosomes also contain multiple activated and functional proteins which could also facilitate the effect of cancer therapy. One study detected high levels of all seven α and seven β chains of the 20S proteasome and three β subunits of the immunoproteasome in MSC-derived exosomes (Lai et al., 2012), implicating a therapeutic potential to target cancer cells by using exosome-delivered proteasomes.

### RNAs

Abundant miRNAs are frequently detected in exosomes isolated from either cell culture medium or bodily fluids (Yu et al., 2015). Most of these miRNAs are functionally involved in exosome-mediated cell-cell communication, and a subset of them exhibited anti-cancer properties. For example, miR-146b-enriched exosomes efficiently transfer miR-146b into glioma cells, inhibiting their proliferation, and reducing glioma xenograft growth in rats (Katakowski et al., 2013). EGFR-specific binding peptide GE11 can guide Let-7a-containing exosomes to EGFR-positive cancer cells, which dramatically inhibited EGFR-positive human breast cancer cell growth in a xenograft mouse model (Ohno et al., 2013). Moreover, exogenous miRNA-143-loaded exosomes significantly reduced osteosarcoma cell migration (Shimbo et al., 2014).

MiR-122-transfected MSCs derived from adipose tissue can produce miR-122 loaded exosomes. These miRNA-loaded exosomes can deliver miR-122 into hepatocellular carcinoma cells to increase their sensitivity to chemotherapeutic agents through altering genes such as cyclin G1, a disintegrin, metalloproteinase domain-containing protein 10 (ADAM10), and insulin-like growth factor receptor 1. Furthermore, miR-122-loaded exosomes dramatically reduced human hepatocellular carcinoma growth in xenograft mice (Lou et al., 2015). MiR-134-enriched exosomes can reduce breast cancer cell migration, invasion, and enhance their chemosensitivity through suppressing transcription 5B, heat shock protein 90, and Bcl-2 (O'Brien et al., 2015). MSC-derived exosomes loaded with anti-miR-9 are able to reverse the expression of multidrug transporters in drug resistant glioblastoma multiforme cells, leading to an enhanced sensitivity to temozolomide treatment (Munoz et al., 2013). In contrast, exosomes derived from docetaxel- or adriamycin-resistant breast cancer cells contain distinct miRNAs which can decrease the drug sensitivity in targeted tumor cells (Chen et al., 2014; Mao et al., 2016). In this case, drug resistance can be transferred from CSCs to other tumor cells. This solidifies the importance of blocking CSC-exosome transfer as an important aspect of cancer therapy.

Using exosomes to silence genes in tumor cells by loading them with siRNAs has been explored in recent years (Alvarez-Erviti et al., 2011; El-Andaloussi et al., 2012). For example, delivery of siRNA against RAD51 via exosomes dramatically inhibited the proliferation of human breast cancer cells and caused their death *in vitro* (Shtam et al., 2013). Exosome-mediated transfer of siRNA against c-Myc can efficiently silence c-Myc and activate the pro-apoptotic protein caspase-3 in mouse lymphoma cells (Lunavat et al., 2016). Exosomes can also deliver PLK-1 siRNA into bladder cancer cells to silence PLK-1, reducing their proliferation (Greco et al., 2016).

Although exosome mediated anti-cancer effects were observed in various tumor models (Table 1), more *in vivo* and clinical evidence for different kinds of tumors are still needed. Therefore, developing more specific CSC targeting exosomes will be a promising cancer therapy strategy in the future.

**Table 1:** **Therapeutic cargos loaded in exosomes for cancer therapy**.

**Cargo**	**Origin of exosomes**	**Target cancer type**	**Loading method**	**Administration route**	**Outcome**	**References**
Doxorubicin	Monocyte or macrophage	Colon adenocarcinoma	Incubation	*i.v*.	Inhibition of tumor growth	Jang et al., 2013
Doxorubicin	Breast cancer cell	Breast and ovarian tumor	Electroporation	*i.p*.	Inhibition of tumor growth	Hadla et al., 2016
Doxorubicin	Immature DC expressing iRGD	αv integrin-positive breast cancer	Electroporation	*i.v*.	Inhibition of tumor growth	Tian et al., 2014
Doxorubicin	Blood	Hepatoma	Incubation	*i.v*.	Inhibition of tumor growth	Qi et al., 2016
Paclitaxel	MSC	Pancreatic adenocarcinoma	Incubation	N/A	Inhibition of proliferation	Pascucci et al., 2014
Paclitaxel	Prostate cancer cell	Prostate cancer	Incubation	N/A	Increased cytotoxicity	Saari et al., 2015
Paclitaxel	Macrophage	Drug resistant cells, lung carcinoma	Incubation, sonication, electroporation	*i.n*.	Overcome drug resistance; inhibition of tumor growth	Kim et al., 2016
Withaferin A	Bovine milk	Breast and lung cancer	Incubation	*i.p*.	Inhibition of tumor growth	Munagala et al., 2016
Celastrol	Bovine milk	Lung cancer	Incubation	*i.g*.	Inhibition of tumor growth	Aqil et al., 2016
hMUC1	hMUC1-expressing carcinoma cell	hMUC1-expressing carcinoma	Pre-overexpression	*i.d*.	Inhibition of tumor growth	Cho et al., 2005
Survivin-T34A mutant	Melanoma cell	Pancreatic adenocarcinoma	Pre-overexpression	N/A	Induction of apoptosis, enhanced chemosensitivity	Aspe et al., 2014
miR-146b	MSC	Glioma	Pre-overexpression	*i.t*.	Inhibition of tumor growth	Katakowski et al., 2013
let-7a	HEK293 cell expressing GE11	EGFR-expressing breast cancer	Pre-transfection	*i.v*.	Inhibition of tumor growth	Ohno et al., 2013
miR-143	MSC	Osteosarcoma	Pre-transfection	N/A	Inhibition of migration	Shimbo et al., 2014
miR-122	MSC	Hepatocellular carcinoma	Pre-overexpression	*i.t*.	Enhanced drug sensitivity, inhibition of tumor growth	Lou et al., 2015
miR-134	Breast cancer cell	Triple-negative breast cancer	Pre-transfection	N/A	Reduced migration and invasion; enhanced chemosensitivity	O'Brien et al., 2015
Anti-miR-9	MSC	Drug resistant glioblastoma multiforme	Pre-overexpression	N/A	Enhanced chemosensitivity	Munoz et al., 2013
RAD51 siRNA	Breast cancer cell	Breast cancer	Transfection, electroporation	N/A	Inhibition of proliferation; induction of apoptosis	Shtam et al., 2013
c-Myc siRNA	Monocytic cell	Lymphoma	Electroporation	N/A	Induction of apoptosis	Lunavat et al., 2016
PLK-1 siRNA	HEK293 cell or MSC	Bladder cancer	Electroporation	N/A	Inhibition of proliferation; induction of apoptosis	Greco et al., 2016

*DC, dendritic cell; MSC mesenchymal stromal cell; hMUC1, human mucin 1; HEK293, human embryonic kidney293; EGFR, epidermal growth factor receptor; PLK-1, polo-like kinase 1; i.v., intravenous; i.p., intraperitoneal; i.n., intranasal; i.g., intragastrical; i.d., intradermal; i.t., intratumoral; N/A, not applicable*.

### Opportunities for exosome-mediated CSC targeting delivery

**Figure 2:**
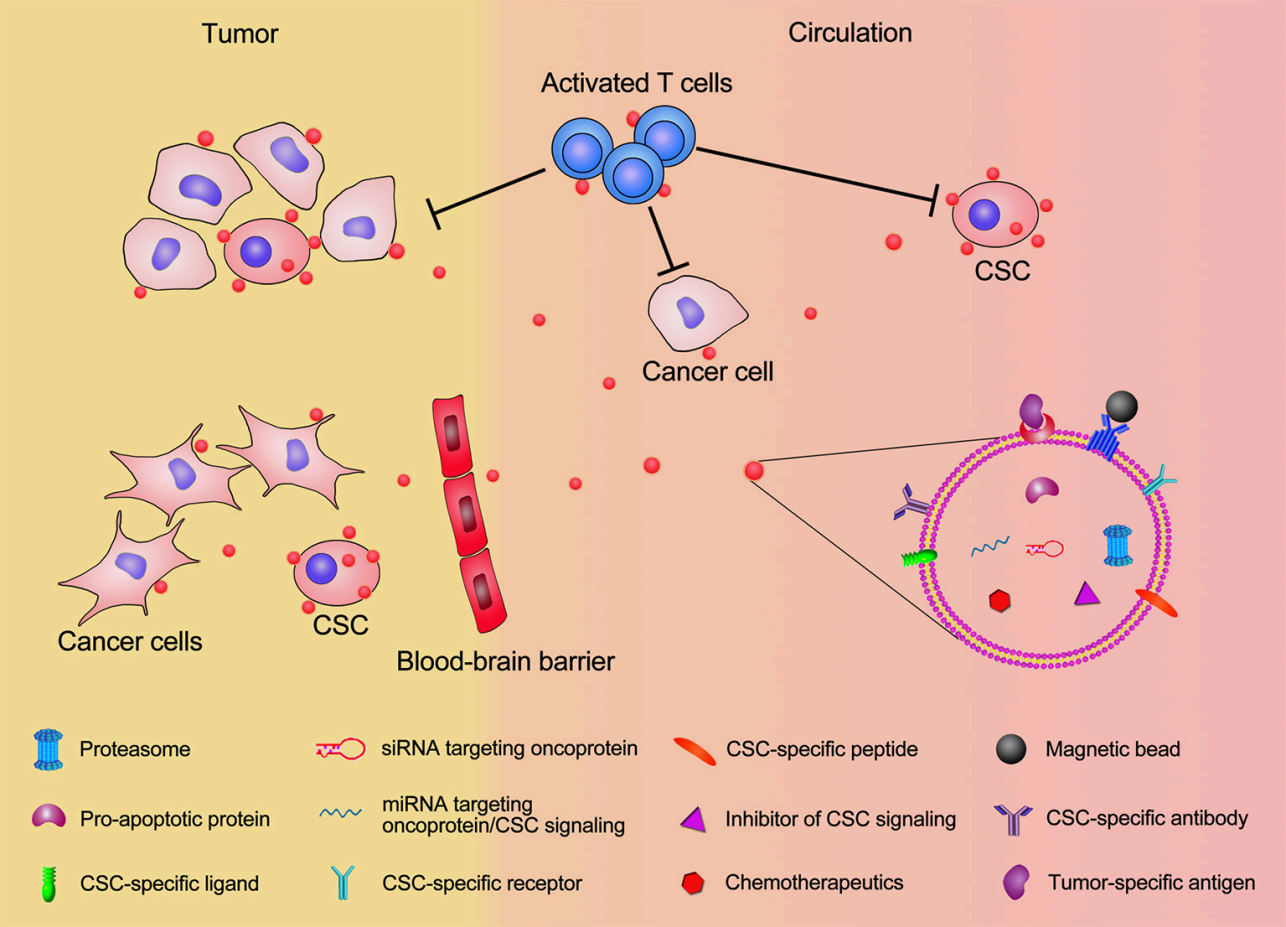
**Schematic illustration of exosomal modification and cargo loading for CSC targeting**. Exosomes displaying CSC-specific peptides, magnetic beads, CSC-specific receptor/ligand, and antibodies for CSC surface maker will significantly enhance the accumulation of exosomes in tumor and increase their CSC targeting specificity. The therapeutic exosome cargos, including chemotherapeutics, inhibitors of CSC signaling, siRNA/miRNA targeting oncoprotien and CSC signaling, pro-apoptotic proteins, and proteasome will elevate the efficiency of killing CSCs. Exosomes present CSC-specific antigens to T cells and activate T cells for anti-CSC immunization. Modified exosome carrying therapeutic cargos can pass through the blood-brain barrier and facilitate CSC targeting in brain tumor.

As described above, exosome-mediated cancer therapy has been widely studied and proved to have great potential for CSC targeting. Using the identified CSC features, we can improve current exosome engineering techniques to allow more precise targeting (Figure 2). The cell surface marker CD44 is highly expressed in high tumorigenic and metastatic hepatocellular CSCs, and Anti-CD44 antibody-coated liposomes can deliver doxorubicin directly to CSCs positive for this marker (Arabi et al., 2015). Interestingly, the anti-CD44 antibody itself can induce the apoptosis of CD90^+^ hepatocellular carcinoma stem cells (Yang et al., 2008). Conceivably, an anti-CD44 antibody-coated exosome could directly induce CSC death, along with their drug delivery role. Therefore, other CSC markers like CD133, CD24, epithelial cell adhesion molecule (EpCAM), and CD200, can also be used as targeting candidates to improve the exosome-mediated CSC targeting efficiency. Since CSC cell surface markers can vary from tumor to tumor, in the future multiple-antibody coated exosomes will need to be engineered to improve their CSC targeting efficiency and to reduce the side effect on normal cells; this because normal cells may present one CSC cell surface marker, but not several of them simultaneously. In addition to targeting cell surface markers, exosomes can also target CSC specific signal pathways. For example, Wnt, Notch, Hippo, Hedgehog, NF-κB, and TGF-β pathways are crucial to maintain the CSC capacities such as self-renewal, differentiation, tumor initiation, and drug resistance (Dandawate et al., 2016; Huang and Rofstad, 2016; Rinkenbaugh and Baldwin, 2016). Using exosomes loaded with inhibitors, miRNAs, or siRNAs to target these pathways can be considered an alternative way to achieve CSC targeting.

### Clinical trials of exosomes in cancer therapy

Since many promising results have been achieved *in vitro* and in animal models, using exosomes for CSC targeting is considered to be one of the most hopeful approaches for cancer treatment. Notably, some clinical trials have already made important achievements (Table 2). As showed in a phase I trial, metastatic melanoma patients were intradermally and subcutaneously given exosomes obtained from autologous DCs and loaded with the MAGE tumor antigens for 4 weeks. Although no significant outcome has been observed yet, the safety and feasibility of exosome administration have been confirmed in these patients (Escudier et al., 2005). Another phase I trial showed that the immune response was activated and disease progression was slowed in a small number of exosome-treated non-small cell lung cancer patients (Morse et al., 2005). Evidence showed that exosomes with interferon-γ (IFN-γ) treatment have enhanced immune activation and tumor suppression effects (Viaud et al., 2011). Based on these phase I and preclinical results, a phase II trial was performed which showed that IFN-γ-DC-derived exosomes were capable of boosting NK cell-mediated anti-tumor immunity in advanced non-small cell lung cancer patients. Thirty two percent of participants experienced stabilization for more than 4 months, although the primary endpoint has not yet been reached (Besse et al., 2016). An ascite-derived exosomes combined with GM-CSF treatment was revealed in a phase I clinical trial for colorectal cancer, showing the induction of beneficial tumor-specific antitumor cytotoxic T lymphocyte response (Dai et al., 2008). Another ongoing phase I clinical trial is trying to determine the ability of plant exosomes to deliver curcumin to colon tumor (NCT01294072, http://www.clinicaltrials.gov).

**Table 2:** **Human clinical trials of exosomes in cancer therapy**.

**Cargo**	**Origin of exosomes**	**Cancer type**	**Phase**	**Results**	**References**
Tumor antigenic peptides	Dendritic cells pulsed with antigenic peptides	Melanoma	I	Proof of Feasibility and Safety; toxicity < Grade II	Escudier et al., 2005
Tumor antigenic peptides	Dendritic cells pulsed with antigenic peptides	Non-small lung cancer	I	Proof of feasibility and Safety; toxicity < Grade I-II, 9/13 completed therapy	Morse et al., 2005
Tumor antigenic peptides	IFN-γ-matured dendritic cells pulsed with antigenic peptides	Advanced non-small cell lung cancer	II	32% of participants experienced stabilization for more than 4 months; boosted NK cell-mediated anti-tumor immunity	Besse et al., 2016
	Autologous ascites	Colon Cancer	I	Proof of feasibility and Safety; Toxicity < Grade I–II	Dai et al., 2008
Curcumin	Plant	Colon Cancer	I	Ongoing	NCT01294072

Overall, multiple clinical trials are ongoing. In the future, standardization of the exosome isolation, storage, cargo loading, quality control, and efficacy evaluation procedures will be necessary for clinical trial and treatment.

### Perspective

Exosomes, as a natural nanocarrier, have great potential as a cancer therapy; however, more work is still needed, especially for *in vivo* studies and clinical trials. Exosomes derived from cancer cells carry functional cargos which directly or indirectly facilitate tumor cell growth (Wang et al., 2014, 2015, 2016a). Therefore, how to identify and remove those tumor supporting components from exosomes is critical for exosome-mediated cancer therapy, and efforts to improve the cargo loading efficiency of exosomes should be a focus going forward. Currently, electroporation is still the best way for loading siRNAs, miRNAs, and small DNA fragments into exosomes, unfortunately this process often induces their aggregation and degradation (Kooijmans et al., 2013). An improved method for sensitive cargo loading, such as siRNA and mRNA, is urgently needed. Using functional exosomes to facilitate immunotherapy is a promising therapy for cancer treatment, since exosomes are more stable than activated antigen presenting cells and can be easily engineered. These features result in exosome-based delivery systems being one of the best approaches for CSC targeting therapy.

## Author contributions

JW and YZ wrote the manuscript and prepared figures. MZ provided critical comments and revised the manuscript.

## Conflict of interest statement

The authors declare that the research was conducted in the absence of any commercial or financial relationships that could be construed as a potential conflict of interest.
